# Effect of Different Energy Levels of Microwave on Disinfection of Dental Stone Casts

**DOI:** 10.5681/joddd.2013.022

**Published:** 2013-08-30

**Authors:** Mahmood Robati Anaraki, Farzaneh Lotfipour, Elnaz Moslehifard, Ali Momtaheni, Pooyan Sigari

**Affiliations:** ^1^Dental and Periodontal Research Center, Tabriz University of Medical Sciences, Tabriz, Iran; ^2^Assistant Professor, Department of Prosthodontics, Faculty of Dentistry, Tabriz University of Medical Sciences, Tabriz, Iran; ^3^Drug Applied Research Center, Tabriz University of Medical Science, Tabriz, Iran; ^4^Associate Professor, Faculty of Pharmacy, Tabriz University of Medical Science, Tabriz, Iran; ^5^General Practitioner, Private Practice, Sabzevar, Iran; ^6^Post-graduate Student, Department of Oral and Maxillofacial Surgery, Faculty of Dentistry, Kerman University of Medical Sciences, Kerman, Iran

**Keywords:** Disinfection, microwave, stone cast, sodium hypochlorite, sterilization

## Abstract

**Background and aims:**

Current chemical methods may not efficiently disinfect dental stone casts. The aim of this study was to investigate if microwave irradiation is effective for disinfection of stone casts.

**Materials and methods:**

In this laboratory study, three groups (n = 162) of prepared spherical stone beads as carriers with a diameter of 10 mm were inoculated by separately soaking in three broth culture media, each containing a study microorganism—Pseudomonas aeruginosa, Staphylococcus aureus or Candida albicans. Six inoculated carriers were used for every test, including irradiation in a household microwave oven at 300, 450, 600 or 900 W energy level, or soaking in 0.03%, 0.06%, 0.12%, 0.25% or 0.50% concentration of sodium hypochlorite solution, at 1, 2, or 3-minute test times. Positive and negative control groups were considered for each test. All treated carriers were then individually transferred to nutrient broth culture medium and one milliliter from each tube was cultured in nutrient agar media over night. Colony forming unit per milliliter (CFU/mL) was counted, and multi-factor ANOVA was used to analyze data (α = 0.05).

**Results:**

Microwave irradiation at 600 W resulted in high-level disinfection in 3 minutes. Immersion of the stone casts in hypochlorite solution at 0.06% concentration resulted in disinfection after 2 minutes.

**Conclusion:**

According to the results, high level disinfection of the stone casts can be achieved by microwave irradiation at 600 W in 3 minutes, similar to a validated chemical method.

## Introduction

Cross-contamination is a major concern in dentistry, especially in the field of prosthodontics, where stone casts become potential sources for transmission of infection from the clinical setting to the laboratory area and vice versa.^1,2^ Appropriate measures, therefore, should be adopted to avoid this kind of cross-contamination. Porous and extremely water-absorbing nature of gypsum casts make them extremely susceptible to contamination, and surface disinfection is inadequate because of deep penetration of microorganisms.

Numerous studies have been carried out to find a safe and effective disinfection technique for stone casts, which mostly concentrate on a range of techniques from using chemical disinfectants as ingredients of the impression and gypsum materials or adding disinfectants during stone mixing,^3-6^ to disinfection of the impressions and poured casts. However, except for the disinfection of the poured cast, none of the explored methods are able to prevent the repeated chances for cross-contamination. The chemical disinfection of gypsum casts is extremely limited due to human health and environmental concerns.^7,8^The daily preparation of fresh disinfection solutions as well as soaking and drying of the casts is costly and time-consuming. Furthermore, apart from having a bad odor, resistance of new pathogens to chemical biocides,^9^ and the negative effects of these materials on physical and mechanical properties of stone casts are among other disadvantages.^10,11^

An interest towards physical disinfection techniques has grown recently, with microwave irradiation applied in dentistry,^12,13^ and other similar areas.^14^ Microwave irradiation has proved effective in sterilization of acrylic resin samples and dentures. In addition, other studies have shown sterilization and disinfection of stone casts using high-level microwave energy that is within the safe range in terms of the tensile strength of stone casts;^15,16^ however, other studies on drying the gypsum casts show that the mechanical and especially the physical properties can be preserved only in low-to-medium levels of energy, and the higher energy levels have a detrimental effect on the cast.^17-19^

In a similar context, medium-level microwave irradiation has also prevented denture-originated cross-contamination.^12,13^ If gypsum casts can also be disinfected at this energy level, this technique could be applied in all stages of clinical and laboratory procedures with a household microwave oven as a practical and safe disinfection method, without the complexity and disadvantages of chemical and high energy level irradiation techniques. Therefore, the aim of the present study was to investigate the effectiveness of disinfection using different energy levels of microwave irradiation on contaminated stone casts with three test microorganisms in comparison with a validated chemical technique according to European Standard EN 1040.^20^

## Materials and Methods

### Sample Preparation and Aseptic Procedures

**Figure 1:**
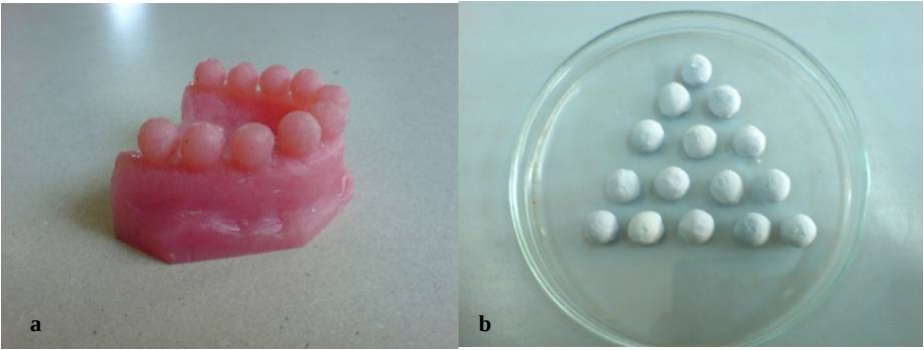
(a) acrylic model prepared to make identical specimens, (b) stone specimens after separating from the cast.

Acrylic dental arches were prepared with 10 identical spherical extensions of 10 mm in diameter in place of teeth. Then, stone casts were prepared by duplicating the acrylic models using an irreversible hydrocolloid impression material (Tropicalgin, Zhermack, Italy) and a Type III dental stone (Elite Model, Zhermack, Italy), both mixed with sterile distilled water according to manufacturer’s instructions. The casts were separated from the impressions 1 hour after pouring and left for 2 hours at ambient conditions. Then, the spherical stone extensions were separated from the casts by a sterile rongeur. The identical spherical stones would hold the same microbial loads, making them suitable carriers to be used as test and control samples (Figure 1). The stone and irreversible hydrocolloid impression powders were not sterilized and were readily dispensed from original manufacturer’s packages. The acrylic models, trays, bowels, spatulas, and rongeurs were disinfected by 70% ethanol.^15^

### Bacterial Strains and Chemical Disinfectant 

Standard strains of Pseudomonas aeruginosa (ATCC: American Type Culture Collection 9027) as a persistent species and Staphylococcus aureus (ATCC 6538) as vegetative bacterial strains, as well as Candida albicans (ATCC 10231) as a fungus were used in this study. The strains were obtained in lyophilized form (Pasteur Institute, Tehran, Iran) and were activated by culturing in Luria Bertani (LB) agar medium (Hi Media, India) and incubated at 37°C for 24 hours. Single colonies taken from the plates were transferred into 4 mL of fluid LB medium and incubated in a shaking incubator at 200 rpm overnight at 37°C. The cells were harvested by centrifugation at 3000 rpm for 15 min at 4°C. Consequently, they were washed twice and resuspended in Ringer solution to provide bacterial concentrations of 10^7^–10^8^ CFU/mL.^21^

Sodium hypochlorite (Pakshoo, Tehran, Iran) was supplied as a stock product, from which dilutions of 1:10, 1:20, 1:40, 1:80 and, 1:160 (with 0.5%, 0.25%, 0.125%, 0.06% and, 0.03% free chlorine, respectively) were prepared, using sterile distilled water as diluent.

### Contamination of Samples and Antimicrobial Experiences 

The stone samples were inoculated by soaking in broth cultures containing 10^7^-10^8^ CFU/mL of each microorganisms for 15 min, each yielding approximately 5×10^6^ CFU/mL. The carriers were removed with a hooked needle and allowed for surface water to evaporate for 2 min in Petri dishes matted with two paper filter sheets.

Half of the inoculated carriers were separately placed in a microwave instrument (Samsung PG 3210, China) at energy levels of 300, 450, 600 or 900 W at 2450 Hz for 1, 2 or 3 min. Simultaneously, a container of water was placed in the microwave chamber to protect the magnetron against excessive energy which might be produced after all the moisture of the samples was evaporated.^18^

The other half of the inoculated carriers were separately placed in the sodium hypochlorite solution at 1:10, 1:20, 1:40, 1:80 or 1:160 concentrations for 1, 2, or 3 min. Subsequently, the samples were carefully removed and placed for 20 min in sterile tubes containing 10 mL of neutralizing broth (Letheen Broth/Difco, USA). Efficacy of the inactivation fluid (Neutralizing Broth) was verified by performing parallel counts of positive controls.^22^

**Figure 2:**
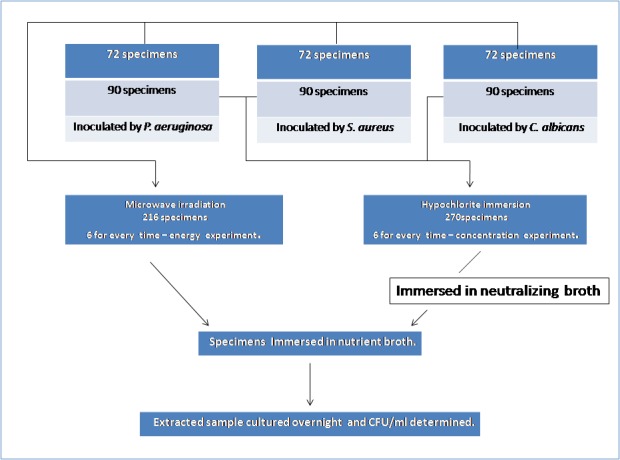
Design of the experiment.

An overview of the study design is given in Figure 2.

### Culturing and Colony Count Procedures

All samples were individually transferred to the sterile tubes with sterile nutrient broth culture media. The tubes were carefully shaken to release the attached bacteria; then samples from each tube were transferred into two 20-cm Petri dishes, one milliliter for each, and topped up to 20 mL with nutrient agar culture media (pour plating method). Each experiment was performed on three separate stages by two samples in every concentration-time and energy level-time composition. After 24 hours of incubation at 37°C, the colonies were counted for every dish and the mean count (MC) of six samples were recorded in colony-forming units per milliliter (CFU/mL). The microbicidal effect (ME) was calculated by subtraction of the log_10_ value of the counts after exposure to the test biocide from the log_10_ value of the counts without exposure to the disinfection procedures (the positive control). The bactericidal activity or disinfection was considered when at least 5 unit reduction in log_10_ microbial counts of test samples compared with those of the positive control samples occurred (ME ≥ 5).^20^

To control the procedures, two control samples were used during each experiment with different concentration-time and energy level-time composition.^22^ One sample containing microbial inocula was selected as the positive control without being exposed to the disinfection process to test vitality of microorganisms and one sample without microbial inocula and without antimicrobial procedures was selected as the negative control to verify the sterility of the procedures. Multifactor ANOVA was used to analyze the results using SPSS 13 software at a statistical significance level of P < 0.05.

## Results

**Figure 3:**
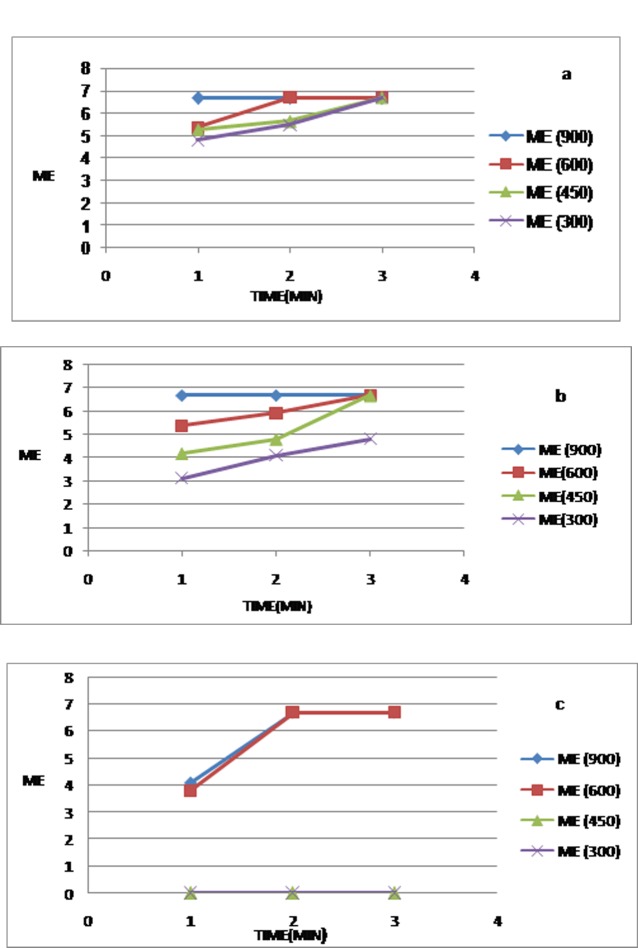
The killing kinetics of microwave irradiation at various energies and exposure times (a: C. albicans; b: S. aureus and c: P. aeruginosa). Microbicidal effect (ME) is log_10_ value of the counts after exposure to the test biocide subtracted from the log_10_ value of the counts without exposure.

The biocidal activity of microwave irradiation at different energy levels and various exposure times for P. aeruginosa, S. aureus and C. albicans are shown in Figure 3. The corresponding counts for the microorganisms are summarized in Table 1. Although microwave irradiation for C. albicans showed disinfection at 300 W after 2 min of exposure, the total killing began after 3 min of exposure at this energy level (MC = 0, ME = 6.69). Experiments carried out on S. aureus–inoculated samples showed disinfection at 600 W within 1 min, with the total decontamination achieved after 3 min at 450 W. However, the total killing (10^6^ log reduction of CFU/mL) of P. aeruginosa was seen at 600 W after 2 min. There were significant differences in MC between microorganisms and energy levels, single or in association together (P < 0.05), but not for times individually (P > 0.05) or in interaction with microorganisms, energy levels, or microorganisms-energy level association.

**Table 1:** Mean counts (CFU/mL) and microbiocidal effect against the microbial and fungal strains

MO/ IT	Untreated	Microwave irradiated							
		900 W		600 W		450 W		300 W	
	MC	ME	MC	ME	MC	ME	MC	ME	MC
P. aeruginosa	5× 10^6^								
1		4.08	407	3.81	776	0	5× 10^6^	0	5× 10^6^
2		6.69	0	6.69	0	0	5× 10^6^	0	5× 10^62^
3		6.69	0	6.69	0	0	5× 10^6^	0	5× 10^6^
S. aureus	5× 10^6^								
1		6.69	0	5.39	.20	4.18	323	3.13	370
2		6.69	0	5.69	10	4.81	.77	4.09	389
3		6.69	0	6.69	0	6.69	0	4.81	.77
C. albicans	5× 10^6^								
1		6.69	0	5.39	.20	5.28	.26	4.8	77
2		6.69	0	6.69	0	5.65	11	5.49	.16
3		6.69	0	6.69	0	6.69	0	6.69	0

MO: Microorganism; IT: Irradiation Time (min); MC: Mean Count; ME: Microbicidal Effect.

**Figure 4:**
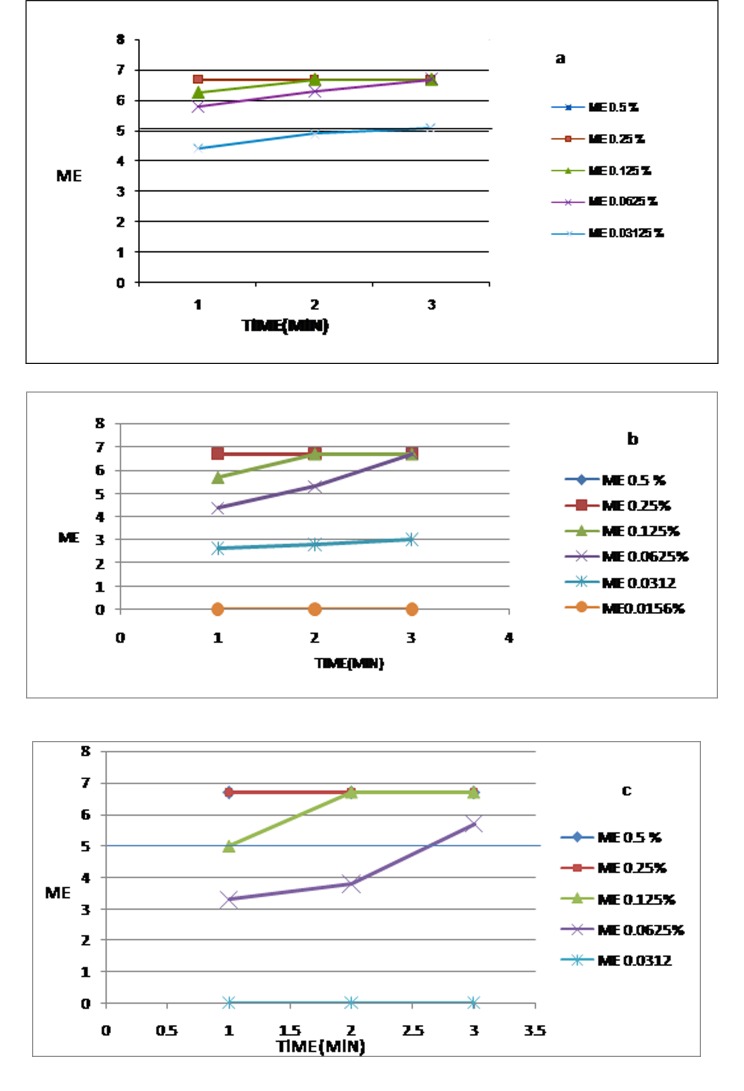
The killing kinetics of sodium hypochlorite aqueous solution at various energies and exposure times (a: C. albicans; b: S. aureus and c: P. aeruginosa). Microbicidal effect (ME) is log_10_ value of the counts after exposure to the test biocide subtracted from the log_10_ value of the counts without exposure.

Table 2 and Figure 4 depict the results of antimicrobial tests from which one can determine the minimum bactericidal concentration of sodium hypochlorite against the selected microbial strains at different immersion times. The samples inoculated with P. aeruginosa and S. aureus were disinfected by 0.062% aqueous sodium hypochlorite after 3 min and 2 min, respectively. The C. albicans inoculuted samples were disinfected in 0.03% concentration after 3 min. There were significant differences in MC between microorganisms and concentrations single or in association together (P < 0.05), but not for times single or in interaction to microorganisms, concentrations or, microorganisms-concentrations association.

**Table 2:** Mean counts (CFU/mL) and microbiocidal effect (ME) of sodium hypochlorite aqueous solution disinfection against the microbial and fungal strains

MO/ IT	Untreated	Disinfected									
		.5%		0.25%		.12%		. 06%		.03%	
	MC	ME	MC	ME	MC	ME	MC	ME	MC	ME	MC
P. aeruginosa	5× 10^6^										
1		6.69	0	6.69	0	4.98	510	3.31	2400	0	5× 10^6^
2		6.69	0	6.69	0	6.69	0	3.81	770	0	5× 10^6^
3		6.69	0	6.69	0	6.69	0	5.69	10	0	5× 10^6^
S. aureus	5× 10^6^										
1		6.69	0	6.69	0	5.69	10	4.39	200	2.61	120×10^2^
2		6.69	0	6.69	0	6.69	0	5.30	25	2.79	81× 10^2^
3		6.69	0	6.69	0	6.69	0	6.69	0	3.01	48× 10^2^
C. albicans	5× 10^6^										
1		6.69	0	6.69	0	6.29	20	5.85	70	4.41	1.94×10^2^
2		6.69	0	6.69	0	6.69	0	6.39	20	4.89	.63× 10^2^
3		6.69	0	6.69	0	6.69	0	6.69	0	5.08	.41× 10^2^

MO: Microorganism; IT: Irradiation Time (min); MC: Mean Count; ME: Microbicidal Effect.

## Discussion

The present study showed contaminated stone casts can be disinfected by a household microwave oven set at 600 W in two minutes. The full decontamination of stone specimens was achieved in three minutes at the same energy level. These results are in accordance with two similar studies by Berg et al,^15,23^showing disinfection of stone casts at 900 W energy level in five minutes, using two of the microorganisms used in the present study. Stone casts have also been sterilized with microwave irradiation at 850 W in 10 minutes, but at the expense of detrimental effects on the physical and mechanical properties.^24^ However, microwave energy levels as low as 650 W have been shown to disinfect stone casts contaminated by oral flora in three minutes.^25^ Therefore, lower levels of microwave irradiation may be advisable for the safe disinfection of stone casts.

Nevertheless, there is controversy over the effect of various energy levels of microwave irradiation on the physical and mechanical properties of stone casts. Low to medium energy levels are suggested to be safer than higher levels by some researchers,^20,21^ while others claim the preservation of certain properties of the stone by drying at energy levels as high as 800 W.^26^ Early irradiation of wet stone casts after pouring results in early drying, thus preventing the completion of the crystallization process and weakening the cast, a fact which is masked by the temporary strengthening as a result of immediate drying caused by irradiation. The assessment of the cast properties after 24 or 48 hours would reveal such severe decrease in the strength of the cast.^17,27^

Chemical disinfection of gypsum casts by immersion in NaOCl solution was used as a standard technique in this study to evaluate the viability and persistence of microorganisms.^28^ In the current study, the disinfection was achieved with regards to P. aeruginosa and S. aureus with the recommended and applied concentration of 1:10 dilution of commercially available NaOCl (approximately 0.06%), similar to that used previously.^29^ Disinfection regarding C. albicans was seen at a lower concentration of 0.03%. The higher persistency of P. aeruginosa to microwave disinfection, similar to that seen in previous literature,^15^ was also seen in the chemical disinfection group.

The disinfection of the stone casts by microwave irradiation can offer a simple, reliable, cost-effective, and non-invasive decontamination method compared to currently available chemical techniques. However, further studies on the physical and mechanical properties of the casts with the recommended microbicidal energy level are warranted to evaluate the effect of repeated microwave irradiations necessary in different clinical and laboratory stages.

## Conclusion

Under the limitations of the present study, it can be concluded that microwave irradiation at 600 W for 3 min can be used to obtain high-level disinfection as a reliable alternative to conventional chemical disinfection techniques.
